# ‘The mothers of England object’: Public Health, Privacy and Professional Ethics in the Early Twentieth-century Debate over the Notification of Pregnancy

**DOI:** 10.1093/shm/hky035

**Published:** 2018-05-31

**Authors:** Salim Al-Gailani

**Keywords:** notification, midwives, pregnancy, privacy, public health

## Abstract

Amid wider efforts to improve maternal and infant health in Britain around the First World War, public health officials debated making pregnancy a notifiable condition. Although the policy never entered national legislation, a number of local authorities introduced ‘notification of pregnancy’ schemes in various guises, with at least one surviving until the 1950s. Resistance from private practitioners to infectious diseases notification in the later nineteenth century has been well documented. We know less about opposition to the extension of this measure to maternal and infant welfare, especially from newly professionalising female health occupations. Conflict over notification of pregnancy drew midwives, in particular, into longstanding arguments over the powers of municipal authorities, family privacy and professional ethics. The controversy was the key battleground in negotiations over the organisation of ‘antenatal care’ as occupational groups of varying degrees of authority sought to define their roles and responsibilities within the emerging health services.

Amid supercharged concerns about ‘national efficiency’ around 1900, health professionals, politicians and activists claimed that pregnancy should be recognised as a ‘State service’ and that all expectant mothers needed access to medical attention at the earliest possible stage.^1^ Introduced among myriad innovations in maternal and infant welfare around the First World War, what came to be termed ‘antenatal care’ can be understood in terms of the numerous turn-of-the-century programmes of social reform that linked norms of bodily management with broader concerns about citizenship and national vitality.^2^ Advocates conceived of antenatal care as the ‘application of preventative medicine to pregnancy’, designed to identify complications and offer advice to the working-class mothers who were the principal target.^3^ This article examines debates around the ‘most pressing problem’ confronting those agencies seeking to build up the new services: ‘linking up … the pregnant woman with the medical profession and with the various institutions’ intended for her benefit.^4^

Report after report on maternity care testified to the difficulty of spreading knowledge that antenatal services existed, and that mothers stood to gain from using them. As wartime conditions prompted experimentation with new infant welfare initiatives, the most widely debated solution was that local authorities should make pregnancy notifiable, like some infectious diseases.^5^ Notification had played a central, if contested, role in the surveillance and control of diseased individuals in Britain since the 1870s. Collective campaigning by Medical Officers of Health (MOHs) had led to the Infectious Diseases (Notification) Acts introduced in England and Wales in the 1880s and 1890s. This legislation represented a significant extension of public health action beyond the sanitary engineering projects of the earlier Victorian era.^6^ Proposals to introduce similar measures for pregnancy followed more directly from efforts to combat the wastage of infant life through the early notification of births. The perceived success of the permissive (1907) then compulsory (1915) Notification of Births Acts, intended to improve both the monitoring of infant mortality and measures to locate and follow up new mothers using health visitors, coincided with a surge of interest amongst MOHs and obstetricians in suggestions that pregnancy, too, should be made a notifiable condition.^7^

Since the measure never made its way into legislation, such proposals might easily be overlooked. Yet the practice was pursued at a local level at a time when much actual policy making and implementation was left to municipal discretion, in many cases driven by an increasingly influential, professionalised corps of medically trained public servants.^8^ These initiatives are still more significant for instigating a debate over the place of the expectant mother within the ‘local welfare state’.^9^ Notification of pregnancy was briefly the key battleground in negotiations over the purpose and organisation of antenatal care as national government, local authorities and voluntary agencies devoted attention and resources to improving the health of mothers and babies. These reforms both facilitated and came to depend upon closer interaction between local health departments, GPs and midwives.^10^ Arguments for and against the notification of pregnancy give us access to demarcation disputes between these various groups as they sought to define responsibilities and manage workloads within a health and welfare apparatus of growing scope and complexity.

Despite the considerable attention given to the development of maternal and infant welfare in Britain, antenatal care remains relatively under researched. Recent histories of municipal provision have emphasised the gradual and localised impact of welfare reforms, which could serve a variety of agendas for different service providers and their clients.^11^ These interpretations have helped to revise older traditions in the historiography that portrayed working-class mothers as passive objects of social control, or focused on direct competition between female midwives and medical men.^12^ But while local case studies have greatly complicated our picture of municipal medicine, antenatal services have thus far escaped significant analysis. This is because antenatal care developed both unevenly and incrementally and was both weakly institutionalised and ambiguously defined well into the interwar period. It was therefore typically less conspicuous in municipal maternity and infant welfare schemes than such better-known services as health visiting. Moreover, antenatal care has been relatively marginal within histories of midwifery, which have tended to concentrate on the impact of the Midwives Acts of 1902 and 1936.^13^ Little is known about the implications of the new emphasis on antenatal medical supervision, which developed largely outside the parameters of national legislation, for midwives and their relations with local health officials.

By focusing on contestation over the notification of pregnancy between the First World War and the 1936 Midwives Act, this article explains how antenatal care fitted into the contemporary practice of public health and was both shaped by and helped to shape wider struggles over professional authority and municipal interventionism. The expansion around 1900 of municipal health provision, and with it the proliferation of salaried roles in public service, exacerbated pre-existing tensions between occupational groups, and between public health and private medicine over the intimately intertwined matters of professional ethics and autonomy. Historians have long recognised that infectious diseases notification posed dilemmas for family doctors, who resisted the measure on the grounds that handing sensitive information to local officials compromised their confidential relationship with their private patients. Moreover, questions of medical confidentiality remained central to debates over the notification of venereal disease.^14^ As Graham Mooney and Tom Crook have recently suggested, the antagonisms the measure provoked in the late nineteenth century were integral to the process through which ‘modern’ public health was assembled and legitimised.^15^ But while previous accounts have concentrated on GPs, this article also examines the implications of notification for recently professionalised, comparatively lowly and, crucially, female occupations.^16^ Conflicts over antenatal care are significant not simply because they revived earlier disagreements over notification, but also because, perhaps for the first time, they drew midwives and health visitors into this complex politics of privacy, paperwork and professional ethics.

Recovering these previously neglected arguments in journals, official circulars, and in the records of professional bodies and government departments, reveals the variety of opinions held by health officials, medical practitioners and midwives concerning the development of municipal antenatal services. It also allows us to examine how the advent of a more ‘feminised’ public health system brought new dimensions to longstanding debates over the balance of personal privacy, professional autonomy and public good. Especially at stake was the status of the midwife, whose involvement in preventive medicine often proved controversial. Anne Hanley’s recent work, for instance, has documented the efforts of medical practitioners to restrict midwives’ participation in the diagnosis and treatment of *ophthalmia neonatorum*, which was made the first notifiable venereal condition in 1914.^17^ The development of antenatal care stimulated similar tensions as midwives and GPs sought to consolidate their respective spheres of professional authority and practice. Yet there was also common ground over the notification of pregnancy, which both groups could claim to be emblematic of an excessive local interventionism that put family privacy and professional autonomy under threat. Among midwives, such matters primarily concerned a relatively small, elite group within the profession who were particularly committed to preserving the status of the midwife as an independent practitioner. By drawing on and extending established medical arguments against notification, these leading midwives were nevertheless significant contributors to a debate that would enduringly shape the principles around which antenatal care was organised and delivered.

## Expectant Mothers and the Local Welfare State

By around 1900, obstetric specialists in various countries were contending that women and their unborn infants could be safeguarded from the risks of childbearing through medical supervision and good hygiene during pregnancy. These arguments fed into a transnational debate about the necessity of public assistance and protection for mothers, including maternity benefits and other ‘family’ welfare reforms. In Britain, provision for expectant mothers developed piecemeal within a ‘mixed economy of welfare’.^18^ A few maternity charities provided for pregnant women in limited ways, including medical treatment, health advice and, in some cases, material aid. Serious interest in the supervision of pregnancy, especially of working-class mothers, only began during the First World War as babies came to be represented as a vital national resource, and as medical practitioners, politicians and activists pressed for more comprehensive protection of maternal and infant health. But even as government activity in maternity and child welfare intensified, the terminology and practice of what ultimately came to be called ‘antenatal care’ remained unstable and contested.

In Britain, antenatal medical supervision was most strongly identified with the Scottish obstetrician John William Ballantyne and his work at the Edinburgh Royal Maternity Hospital, which established the first ‘pre-maternity ward’ for the systematic investigation and treatment of illnesses associated with pregnancy in 1901. Ballantyne promoted an explicitly anti-eugenic ‘antenatal hygiene’ that emphasised the importance of environmental conditions and medical care of the mother during pregnancy over hereditarian ‘fatalism’. As concerns about infant and neonatal mortality and national efficiency intensified in the aftermath of the Boer War, individuals and groups active in the maternal and infant welfare movement embraced antenatal hygiene as a ‘gospel of hope’.^19^

The provision for expectant mothers that emerged in the first two decades of the century is best understood in terms of the growing interpenetration of public and private welfare initiatives. Antenatal hygiene provided the scientific underpinnings for the campaign by women’s and labour organisations, notably the Women’s Co-operative Guild, for a coordinated scheme for the national care of motherhood.^20^ Early practices of antenatal medical supervision were developed by ‘rescue’ charities for unmarried mothers.^21^ But the most significant proselytisers were a generation of influential public health officials who viewed antenatal hygiene as a weapon in the service of preventative medicine. Interest in expectant mothers developed steadily among MOHs and voluntary agencies asserting the importance of ‘antenatal influences’ on infant death and disease.^22^ From around 1913, Arthur Newsholme, chief medical officer to the Local Government Board (LGB), sought to encourage and coordinate the still piecemeal welfare activity targeting mothers and babies. Keen to extend the powers of local authorities in this area, Newsholme made the case for incorporating antenatal hygiene into the ‘administrative machinery’ of infant welfare.^23^

Official reports and memoranda outlining how local authorities might use government grants to develop unified maternity and child welfare schemes enshrined the core principle of statutory antenatal supervision: that ‘medical advice and, where necessary, treatment should be continuously and systematically available for expectant mothers’.^24^ The LGB’s template for antenatal provision combined in-patient pre-maternity care for complicated cases with a system of home visiting and out-patient clinics available to any pregnant woman, whether applying to a hospital for relief or booking her confinement with a midwife or doctor. Antenatal clinics hosted by voluntary hospitals and municipal maternity centres grew in number from 1915, made possible by matching government grants to local authorities. As would be repeatedly stressed, the success of these services relied on a high degree of cooperation between local health authorities, voluntary associations, midwives, GPs and hospitals. It also required occupational groups with distinct interests to recognise the supervision of expectant mothers as a collective endeavour.^25^ But questions about responsibility for antenatal supervision persisted even as expenditure on maternity and infant welfare expanded during wartime.

Policy makers and practitioners alike were suspicious of active state involvement, and significant disagreement remained about the respective duties of obstetricians, GPs and especially midwives. The 1902 Midwives Act introduced arrangements for certifying and regulating midwives’ practice under the new Central Midwives’ Board (CMB), controlled by the medical profession. Intended to develop a formally educated and professional workforce of birth attendants, the Act created the conditions for much closer engagement between local authorities and midwives, increasingly represented as agents of hygiene and social reform.^26^ The responsibility of midwives for antenatal work was formally outlined in the rules of CMB from 1916.^27^ Yet health workers continued to disagree over the value and content, and even the novelty, of antenatal care. Sceptics argued that examining every expectant mother would mean ‘a great deal of unnecessary trouble’ and, since the causes of early infant mortality were known to be so complex, probably for little return.^28^ Midwives could still complain in 1917 that ‘it was impossible … to lay down an absolute definition [of antenatal care] inasmuch as the medical profession, who are the teachers in the matter, have not done so, and in fact differ considerably in their teaching’.^29^

Even the keenest proponents of antenatal supervision acknowledged that this was ‘amazingly difficult work to develop’. The new services were ‘partial and incomplete’ and suffered from ‘lack of coordination’.^30^ Rural areas and smaller provincial towns presented different challenges from more populated urban centres with active voluntary agencies, maternity institutions and trained midwives. Metropolitan hospitals with established out-patient maternity departments could rely on almoners to recruit expectant mothers.^31^ But officials almost everywhere complained about the difficulty of spreading the word that antenatal services existed and convincing women to use them.^32^

Expectant mothers were presumed ‘shy’ or resentful of medical ‘inquisitiveness’ and allegedly disliked attending crowded clinics. For working-class women, in particular, open discussion of pregnancy was considered indelicate, and municipal services were problematically associated with the Poor Law.^33^ The apparent ambivalence towards antenatal care fed into the wider trope of maternal ignorance. Although proponents of antenatal care accepted that inadequate attention to the hygiene of pregnancy was an issue at all levels of society, working-class mothers were the primary targets of such criticisms.^34^ However poor attendance was explained, those promoting and administering antenatal services recognised the need for a coordinated strategy for getting in contact with mothers.

## Should Pregnancy Be a Notifiable Condition?

Notification was by this time established as an instrument of public health. Compulsory infectious diseases notification had previously faced considerable opposition from the private medical profession. Mandatory reporting not only offended deeply engrained notions of medical confidentiality, it also put GPs partly under the scrutiny of the local public health authority, the MOH, who might be a professional rival. By around 1900, however, there was broad agreement that systematic surveillance was necessary for an informed preventative policy. This rested on the consensus that the well-being of society—specifically, freedom from illness—required some infringement of individual liberty.^35^ But disagreements resurfaced as sanitary authorities pressed to extend the principle of notification not just to other diseases, but also into other realms of public health.

Growing concern about the health of mothers and babies turned notification into an administrative tool of infant welfare.^36^ Two issues commanded particular attention. First came the need for improving procedures for the early reporting of births. In England, the law allowed for forty-two days to elapse before a birth was registered. Consensus about the desirability of better information about infant mortality led to the 1907 Notification of Births Act. This legislation empowered local authorities to require those in attendance at a birth to notify the local health authority within thirty-six hours.^37^ This dovetailed with growing pressure for state intervention in the control of venereal disease for the first time since the repeal in 1886 of the controversial Contagious Diseases Acts.^38^ Awareness of the devastating impact of VD upon family and ‘racial’ health was a key strand of the successful campaign by medical, feminist and social hygiene activists for a wider government enquiry into the prevalence and prevention of syphilis and gonorrhoea. The resulting Royal Commission on Venereal Disease, established in 1913, propelled long-standing and divisive questions about the legitimacy and effectiveness of notification to centre stage.^39^

The Royal Commission on VD, followed by the passing of the compulsory Notification of Births (Extension) Act in 1915, prompted the first extended discussions of the potential benefits of making pregnancy similarly notifiable. The case for notification came initially from obstetricians, and particularly those involved with the development of hospital-based antenatal clinics and VD services, both in testimony to the commission and at professional meetings.^40^ By early 1916, the most prominent advocates were MOHs, especially those in urban authorities with comparatively well-developed infant welfare schemes. These proponents presented the notification of pregnancy as a necessary extension of municipal infant welfare.

For both groups, a formal system for notifying pregnancy offered an obvious solution to the challenge of connecting antenatal services with users, and particularly those difficult-to-reach uninsured mothers assumed to be the most in need of medical care. But advocates claimed further benefits for notification, notably that it would enable the collection of more reliable statistics about the incidence of miscarriage and stillbirth, and stimulate wider medical and political interest in the pathologies of pregnancy. Information furnished by notification, some argued, would also equip authorities to tackle the problem of criminal abortion.^41^ Proponents also maintained that the policy would aid efforts to improve procedures and facilities for the investigation and treatment of VD, including understanding of the consequences of syphilis and gonorrhoeal infection on the fetus.^42^ In doing so, they articulated the need for cooperation between emerging antenatal and VD treatment services, which by the end of the war were becoming more closely—if in practice rarely straightforwardly—integrated.^43^ As one health official put it, notification would ‘make possible the efficient surveillance and supervision’ of those pregnancies most in need of medical attention.^44^

Other obstetricians and MOHs were more ambivalent about the notification of pregnancy. Given that diagnosing early pregnancy was notoriously difficult, cynics were doubtful as to the practicality and value of the measure. Most concerns focused on the possible deterrent effect of notification. Unmarried mothers were presumed especially unlikely to seek help and advice if it led to the involvement of a public official, however well intentioned. Even married women were ‘inclined to be reserved’ about their pregnancies and the stigma of publicity was likely either to ‘frighten off shy mothers’ or discourage them from engaging a doctor or midwife until the last moment.^45^ Even those who accepted the benefits acknowledged that a public ‘ready conscientiously to object’ to vaccination and controls on VD was unlikely to embrace the notification of pregnancy.^46^

Despite these concerns, three potential models for notifying pregnancy had entered public debate by the First World War. The most radical calls were for a compulsory system obliging all expectant mothers to declare their pregnancies to an official.^47^ Such proposals went against the grain of medical opinion, which tended to dismiss any form of coercion as counterproductive. In the case of VD, medical practitioners typically argued against compulsion on the grounds that the stigma of official notification would deter sufferers from seeking medical help.^48^ Similarly, even those disposed to make pregnancy notifiable conceded that expectant mothers were less likely to seek early medical attention under compulsion.^49^

The second, voluntarist, model proposed encouraging mothers to declare their pregnancies to a local official through a combination of education and pecuniary benefits. Those advancing this approach linked the question of notification to debates over the ‘endowment of motherhood’ and, more specifically, the handling of sickness claims by pregnant women under the 1911 National Insurance Act.^50^ Advocates suggested incentivising early reporting of pregnancy by making adjustments to the maternity benefit, including by making payment dependent on antenatal notification. Such an arrangement would mean transferring responsibility for the maternity benefit to local authorities, as proposed by such figures as the Fabian Sidney Webb.^51^ However, there was little political will to implement any substantive changes to maternity insurance, even at the height of wartime debate over women’s entitlement to social welfare. Benefit reforms were all the more unlikely in a post-war policy climate highly sceptical of arguments that motherhood should be the direct financial concern of government.^52^

Objections to the first two models for notifying pregnancy did not, however, rule out a third. This would appeal especially to MOHs, seeking to consolidate their professional influence and extend the remit of their departments at a time of unprecedented consensus about the need for a comprehensive community health strategy.^53^ In the absence of any financial inducements to encourage women to declare their own pregnancies, some MOHs proposed that notifications might be secured by incentivising not the mother, but the birth attendant. This would require a midwife or doctor, on taking a booking, to register the details of the client with the local authority for a fee.^54^ This had the advantage of keeping the question of antenatal provision separate from health insurance, and within the sphere of municipal health departments, already responsible for the administration of the Midwives Act, and recently empowered by the Notification of Births (Extension) Act to develop services for expectant mothers. Such arrangements remained open to interpretation, and some MOHs regarded notification of pregnancy as a means of tightening administrative control over what they perceived as a fragmented and contested maternity and infant welfare apparatus.

Despite the emergence of maternal and infant welfare as a priority for both national and local government, policy innovations in this area were met with caution, and often hostility, from the private medical profession. Such attitudes built on longstanding objections to government involvement in health care, which had resurfaced in debates surrounding the 1911 Insurance Act.^55^ Notification of births had been unpopular with GPs, on whom it imposed the obligation of reporting promptly all births that they had attended at risk of penalty. Although some resistance to notification of births remained at least until the Extension Act of 1915, by wartime direct municipal involvement in the maternity services had become the main focus of GPs’ grievances.^56^ These were given expression by the British Medical Association (BMA), which warned members that impending legislation in this area proposed to ‘vitally alter the present status of the profession’ with potentially ‘disastrous’ consequences for GPs. As a professional body representing private practitioners’ interests, the BMA reckoned municipal maternity clinics undermined the status of the family doctor. The clinics not only impinged on the right of patients freely to choose their medical attendant, but also threatened to destabilise GPs’ economic position by losing them fee-paying patients.^57^ News that a number of MOHs were advocating for notification of pregnancy only sharpened these concerns.

In a highly critical commentary on maternity and infant welfare, the *British Medical Journal* singled out the prospect of notification of pregnancy as particularly dangerous for the private practitioner. Such a policy would effectively hand the ‘“tied” doctor, the servant of a [sanitary] authority’, unfettered access to the expectant mother, the traditional ‘gateway’ to family practice. The CMB already stipulated that midwives refer sickly pregnant women to a doctor. Notifying cases to the local authority the editorial persisted, was therefore simply unnecessary. Such interference would disrupt relations between midwives and the medical profession, whose work spheres were regulated by the CMB. It would also ‘sicken [mothers] of medical examination’, heighten distrust of doctors and midwives and, consequently, ‘much needed treatment will go by default’.^58^

By summer 1916, a number of local divisions of the BMA had drawn the attention of the Representative Council ‘to a movement in favour of notification by doctors and midwives of pregnancy’, even though barely half a dozen local authorities had by then taken any steps to introduce the practice.^59^ The medical press had by then reported on attempts to introduce the measure in Huddersfield, Chatham, Nottingham, St. Helen’s, York and at least two London boroughs.^60^ For these largely urban MOHs, notification of pregnancy was a means of aiding cooperation between their department and the various health practitioners involved with maternity care, securing access to expectant mothers and identifying those in need of medical attention. An obstetrically-trained medical officer employed by the local authority, they argued, was more capable of diagnosing pathologies than a midwife or GP.^61^

While the BMA was ‘entirely in favour of any scheme which will encourage prospective mothers to arrange in advance for their nursing and medical attendance’, it advised that notification of pregnancy would have ‘quite the contrary effect’. Drawing on arguments previously deployed against notifying VD and births, it was suggested that all mothers, and especially the unmarried, would inevitably ‘fight shy of consulting either a doctor or a midwife until the last available moment, for fear that their condition be made more or less public’.^62^ Notifying births to a public authority, some GPs had previously claimed, prevented ‘respectable’ families from concealing the ‘shame’ of an illegitimate pregnancy.^63^ With the profession openly debating such concerns, notification of pregnancy could be represented as a still worse violation of family privacy.

At the Annual Representative meeting in summer 1916, the BMA carried a motion formally opposing the notification of pregnancy.^64^ This stance ruled out any prospect that the LGB might recommend the practice more widely. In a previous report to the LGB, Newsholme had cautiously endorsed notification of pregnancy by birth attendants.^65^ However, he now accepted that this ‘somewhat indiscreet’ measure could only ‘prejudice the end to which it is directed’, namely antenatal supervision. The official line was now that ‘formal notification of pregnancy should not form part of a maternity and child welfare scheme’.^66^

For those promoting antenatal supervision, then, the war and post-war years were an opportune time to advance arguments about the benefits of making pregnancy notifiable. Medical and political investment in midwifery reform, infant mortality, VD and maternity benefit changes all contributed to the consolidation of notification as an instrument of infant welfare, and a potential solution to the specific challenges of antenatal care. These proposals not only revived longstanding grievances about notification, but also gave expression to professional struggles within the maternity services.

## Bullies, Bribes and Busybodies

From the perspective of the LGB, robust resistance from the BMA made a formal system for notifying pregnancy politically undesirable as well as impractical. But even as Newsholme rejected as ‘erroneous’ arguments that the practice was ‘a necessary antecedent to the commencement of an antenatal clinic’, he persisted in leaving open the option of notification by midwife. The MOH had no authority over GPs, but did have a key role in the administration of the Midwives Act, which placed midwives under the official supervision of the municipal council (as local supervising authority). MOHs, Newsholme suggested, could therefore instruct midwives to refer their clients to the municipal maternity centre, so long as they had the mother’s ‘formal and intelligent consent’.^67^ Disagreement over the interpretation of this recommendation by MOHs erupted into fierce controversy over the autonomy of the midwife, and the encroachment of ‘officialdom’ into the lives of the poor.

Midwives, according to official estimates, still attended the majority of childbearing women in England and Wales, approximately 51 per cent in London and 69 per cent in the county boroughs.^68^ The impact of maternity and infant welfare reforms on the practice, recruitment and social profile of midwives was partial and contingent, especially since it is far from clear that the identity of the midwife as an autonomous professional extended beyond a relatively small, urban elite. Nevertheless, midwives’ work was increasingly defined through relations with sanitary authorities.^69^ The growing emphasis on antenatal supervision offered midwives with recognised training the opportunity to augment their public health role and distinguish themselves from untrained competitors. Yet closer engagement with local authority officials risked compromising midwives’ independence.^70^ This dilemma lay at the heart of midwives’ concerns over the notification of pregnancy.

The prospect of notification was challenged most actively by the leadership of the London-based pressure group, the Midwives Institute, and particularly the influential lobbyist Rosalind Paget, who held the post of treasurer until 1930. Formed in 1881 to raise the status of midwifery as an independent profession, the Institute was dominated by upper-middle class metropolitan women, and historians debate the extent to which it represented the overwhelmingly working-class and provincial rank-and-file.^71^ While there is evidence of broad-based opposition to the notification of pregnancy, the campaign was undoubtedly spearheaded by an elite for whom professional demarcation disputes mattered most. The leadership of the Institute lobbied against notification, both in principle and in practice, including by reporting on the actions of local authorities in nursing and midwifery journals and pro-suffrage periodicals, advising regional midwives associations, and by formally petitioning the LGB on their behalf. These efforts were backed by other national bodies representing female-dominated heath professions, the Women’s Cooperative Guild, the BMA and, most significantly, by the Central Midwives Board, on which Paget sat until 1924.^72^

The range of arguments put forward by the Midwives Institute and their allies against the notification of pregnancy operated at a number of levels. The principle of notification was rejected on moral grounds as a violation of personal freedom, and particularly that of the working-class mother; and, so too, on ethical grounds, as threatening the confidential relationship between the midwife and her patients. Paget’s close involvement with the CMB, her commitment to the independent midwife and ambivalence towards the creation of a salaried midwifery service undoubtedly motivated her criticism of the practice. But these concerns encompassed a deeper, and more widely shared, set of fears and aspirations about the social, professional and, ultimately, economic standing of the midwife.

The leadership of the Midwives Institute reacted with ‘puzzlement’ to contradictory official advice. The rules of the CMB entrusted certified midwives with the care of their clientele prior to as well as during their confinements. Yet LGB recommendations about antenatal supervision implied that local authorities should take primary responsibility for this work. Midwives, ‘no less than the medical profession’, the Institute insisted, were bound to treat all information obtained in their ‘professional capacity’ as a ‘sacred confidence’. Yet some supervising authorities were now requesting that midwives divulge these ‘intimate particulars’ to officials.^73^

Critics diagnosed a worrying trend towards local government control over maternity care and the undermining of the midwife’s role. Some authorities, as in Huddersfield, were offering a ‘municipal bribe’ to ‘violate professional confidence’ by sending their clients’ details to local officials. Others were ‘bluffing’ midwives with ‘printed notification forms’ under the pretence that the practice had official sanction.^74^ The concern was that midwives were being intimidated or deceived into not only undermining their standing as a profession through a breach of ethics, but also losing a portion of their work, and potentially clientele, either to a municipal clinic or to a professional competitor ‘who did not tell all her patient’s business to the Town Hall’.^75^ Most galling for midwives was the delegation of responsibility for antenatal supervision to health visitors, who they portrayed as inexperienced and sanctimonious interlopers unpopular with working-class mothers.^76^ Midwifery journals carried a stream of editorials and correspondence condemning the practice as an ‘intolerable interference with the liberty of women’, midwives and expectant mothers alike.^77^

By linking notification of pregnancy to debates over women’s citizenship, leading midwives tapped into wider arguments concerning state intervention in matters of sex. Despite much enthusiasm within the women’s movement for the expansion of maternity and infant welfare, agitation against the Contagious Diseases Acts had given activists a tradition of opposition to the state.^78^ As concerns about VD control measures regained traction during the First World War, writers in pro-suffrage periodicals cast pressure by health officials for the notification of pregnancy as a ‘new menace to women’ designed to resuscitate the coercive ‘horrors’ of the Acts.^79^ Others in the women’s movement bemoaned that notification of pregnancy reflected ‘official desire for tabulation and statistics’ over genuine understanding of the realities of poor women’s everyday lives and material needs.^80^

Paget and her colleagues similarly portrayed notification of pregnancy as a form of ‘cunningly masked’ coercion, contrasting the discretion of the independent midwife with the allegedly unwelcome interference of the municipal health visitor. Leading midwives argued that it was a woman’s right to decide when and to whom to divulge knowledge of her pregnancy, and that the worry of an official visit or the prospect of an encounter with an unfamiliar doctor could only deter mothers from early booking. Especially concerning was the association between the notification of pregnancy and ‘routine vaginal examination’, which recalled the worst excesses of the Contagious Diseases Acts. Midwives warned this could only hinder the progress of antenatal supervision.^81^ For their part, proponents of notification claimed critics were overstating working-class hostility to the ‘kindly solicitude’ of well-meaning municipal intervention and accused midwives of ‘singular shortsightedness’ in pursuing professional interests over the national well-being.^82^ Particular contempt was reserved for the London-based elites of the Midwives Institute for ‘trying to stir up the large army of midwives … to be antagonistic to the useful and diplomatic work being carried out by health visitors and others.’^83^

For critics of notification it was not just that ‘delicacy and reserve’ were the ‘natural’ conditions of pregnancy. The issue was rather the interdependence of privacy and respectability that made the ‘harassing interference’ of ‘inquisitive officials’ in the lives of the poor so repugnant.^84^ Such schemes represented, in extreme form, the encroachment of ‘officialdom’ into the households and communities of the working classes at a time of intense anxiety about the presence of the machinery of government in everyday life. The expansion of public data collection that came with the emerging welfare system in the early twentieth century, David Vincent has noted, raised new questions about the relations of trust between the increasingly private home and an ever-more intrusive state.^85^ Amid wider concerns about the vulnerability of ‘family secrets’ within a welfare bureaucracy of growing complexity, midwives drew attention to the dangers of putting information about individuals’ reproductive lives into public hands. Any such document ‘sent through the halfpenny post and filed at the Town Hall’, midwives warned, was ‘at the mercy of any busybody who chose to unfold it’.^86^ Indeed, midwives in St Helen’s complained that local postmen were reading the notification forms and poking fun at their clientele.^87^

Opponents thus condemned the notification of pregnancy as ‘repugnant to English feelings and ideas’ of liberty, privacy and fairness.^88^ As Paget asserted in a letter to *The Times*, ‘the mothers of England object to it’.^89^ Working-class women had every reason to be suspicious of overbearing councils, not least because officials showed no inclination to intrude into the lives of the ‘better classes’.^90^ Those MOHs demanding notification from midwives were nothing other than ‘complacent and detestable Busybodies … conscious of nothing save their desire to poke their long, thin noses into poor people’s houses and to impose on them conditions and interferences they would not tolerate for their own wives and daughters’.^91^ Paget and her colleagues could thus claim that the independent midwife was best positioned both to promote antenatal hygiene and protect the privacy of her working-class clientele.

For midwives, to an even greater extent than for GPs, professional identity was at stake. Notification of pregnancy schemes ‘destroyed the patient’s confidence in her midwife’ and compromised her freedom to practise independently.^92^ Leading midwives linked notification to medico-libertarian disquiet about the expansion of the state, highlighted working-class antipathy to officialdom and insisted that antenatal supervision fell rightfully within their work sphere. Such schemes, they argued, reduced midwives to municipal ‘informers’ bullied into violating the confidence of their clients.^93^ Notification, activists claimed, would only antagonise the midwives and expectant mothers on whom the success of antenatal care depended.

Agitation by midwives against notification of pregnancy continued through the war, but largely dissipated thereafter. Disapproval from the CMB discouraged local authorities from pursuing the measure.^94^ By now the priorities of the Midwives Institute had shifted to the more pressing problems of midwife shortages, recruitment and remuneration. Growing expenditure on maternity services following the Maternity and Child Welfare Act of 1918 meant that fewer midwives worked independently, and more were employed by local authorities or district nursing associations. This trend was consolidated by the 1936 Midwives Act, which created a salaried domiciliary midwifery service to cover the whole country.^95^ Changes in the occupational landscape mostly laid the controversy to rest. Yet this did not spell the end of notification of pregnancy entirely.

## Notification in Interwar Municipal Antenatal Care

The replacement of the LGB with the Ministry of Health in 1919 briefly raised hopes for the radical re-development of facilities and administrative structures for antenatal supervision and research. However, any prospect that the new Ministry would support nationwide notification of pregnancy ended when proposals circulated by Walter Morley Fletcher, the secretary of the Medical Research Council, were dismissed as administratively and politically unworkable.^96^ Ministry of Health civil servants, like their predecessors at the LGB, continued to regard notification as unnecessary as local authority health services came under greater scrutiny from central government during a period of expansion and consolidation. Officials promoted municipal clinics as the most appropriate site for antenatal care, especially in urban areas, but councils retained the freedom to experiment with different models of provision. With such variation in services, notification of pregnancy remained but one of many strategies pursued by local authorities to reach expectant mothers and encourage cooperation from midwives and GPs.

Antenatal supervision grew in prominence amid heightened concern about maternal mortality during the 1920s and 1930s. Health officials emphasised the importance of ‘judicious propaganda’ to encourage mothers to use municipal health services. Leant weight by the burgeoning ‘mothercraft’ movement, the presumed significance of antenatal hygiene was reinforced by such perceived successes as the ‘Rochdale experiment’, an educational campaign coordinated by the MOH with the support of the local press, churches, women’s organisations and medical and midwives’ associations.^97^ Yet health officials, both local and national, agreed that the success of antenatal care depended most on the support of midwives, who had ‘the ear and friendship of the mother and would get into touch with the pregnant woman earlier than an official’.^98^ Ministry of Health reports and memoranda on the conduct and scope of antenatal clinics urged local authorities to promote ‘close and cordial’ cooperation with midwives, including by providing training and guidance in patient care and record keeping. The ‘personality and tact’ of the medical officer could do much to discourage antagonism. But Ministry officials recognised that midwives sometimes hesitated to refer their clients to a municipal clinic through fear of financial loss should the medical officer recommend hospital confinement. In such cases, the Ministry advised, councils should be prepared to pay compensation.^99^

Local MOHs continued to protest about the challenges of developing antenatal services and that midwives too often neglected or offered inadequate supervision.^100^ Most nonetheless accepted that it was ‘much better to convince the mothers by successful results rather than coerce them through legislation.’^101^ Municipal efforts to introduce notification schemes were in most cases abortive. Experimentation with the measure nonetheless persisted through the interwar period as MOHs sought to develop maternity and infant welfare initiatives with central government grants. The voluntary scheme introduced in Huddersfield in 1916 was by far the best known. Since it survived until 1951, long enough to be evaluated by Ministry of Health officials, the scheme provides more detailed evidence of such arrangements than comparatively short-lived or informal counterparts elsewhere.

Huddersfield was widely recognised as a pioneering centre of infant welfare, having initiated a comprehensive system of notification of births and health visiting through private legislation in 1905. The cornerstone of the Huddersfield system was the employment of medically qualified women as ‘Assistant MOHs’, who were responsible for both heath visiting and the supervision of midwives. Evidence of declining infant deaths, together with the passage of countrywide notification of births legislation in 1907 and 1915, cemented the reputation of Huddersfield’s MOH, Samson Moore.^102^ Moore claimed that the notification of pregnancy, being a natural extension of domiciliary infant visiting, was unnecessarily controversial. By 1916, he had persuaded the council’s lay health committee, keen to publicise its infant welfare work, that the experimental scheme was a worthwhile use of government grants for this purpose.^103^

In Huddersfield’s notification of pregnancy scheme, any midwife or doctor could claim a fee of two shillings and sixpence for supplying the MOH with the names and addresses of their clients, provided they had the mother’s consent. The mother would receive a home visit from an Assistant MOH, who offered no treatment, but could refer all complicated cases to a doctor or the local Infirmary. They could also assign council-employed home helps to women judged to be in particular need of household assistance. In later years, the council also provided expectant mothers with free milk, cod liver oil and sterilised maternity outfits.^104^

Moore insisted that notification with visiting was better suited to Huddersfield, and allowed for more comprehensive antenatal care, than the municipal maternity centres proliferating elsewhere. He in fact actively disapproved of ‘repugnant’ antenatal clinics, which he believed unpopular with mothers and likely to encourage gossip. In Moore’s view, home visiting permitted the Assistant MOHs to assume the capacity of a ‘sympathetic friend’, while at the same time dispelling any suspicion of Poor Law relief. Home visits, he argued, enabled medical officers to discuss confidential matters, offer advice, and report on the mother’s domestic conditions and personal circumstances.^105^ By around 1920, Moore was claiming ‘satisfactory’ progress. He was able to report a steady increase in the proportion of births ‘antenatally notified’: from 11.2 per cent in 1916, rising to 24.1 per cent in 1917 and 38.3 per cent in 1921.^106^ This figure remained roughly stationary until Moore retired in 1930. For although he boasted that notification of pregnancy operated ‘smoothly and agreeably to all concerned’, in practice the scheme was far from frictionless.^107^

Local GPs were still complaining in the 1930s that the very existence of the scheme had deprived them of both antenatal and postnatal work.^108^ Still more problematically, Moore failed to secure the cooperation of the district nursing association, the Huddersfield Victoria Nurses Association (HVNA), whose midwives attended a significant and growing proportion of births in the borough.^109^ In 1920, he led a deputation from the council to make the case for notification of pregnancy at an extraordinary meeting of the association. The governing committee of the HVNA agreed to comply on the conditions that notifications were made only ‘at the wish of the woman herself’ and that all instructions to midwives be sent in writing through the superintendent of the association.^110^ Yet this was no guarantee of cooperation and, in his final report as MOH, Moore complained that HVNA midwives were, ten years on, still refusing to participate.^111^ Relations between the health department and the HVNA improved, however, following Moore’s retirement. Huddersfield’s new MOH, John Gibson, could report in 1931 that, as a result of ‘more intimate cooperation with the midwives’, he had received antenatal notifications for more than half of all births in the borough. This followed a commitment from the council to compensate midwives for loss of earnings when a client was antenatally notified and referred to hospital.^112^

Shortly after Gibson’s appointment, the Ministry of Heath conducted a review of arrangements in Huddersfield as part of a nationwide survey of public health services following the Local Government Act of 1929.^113^ The Ministry was encouraged by Gibson’s efforts to mend relations with local midwives, among other administrative improvements. There was less enthusiasm, however, about the notification of pregnancy scheme. Ministry officials, notably Janet Campbell, the Senior Medical Officer for maternal and infant welfare, were disparaging of Huddersfield’s ‘unorthodox’ approach. While the infant mortality rate in the borough had fallen in line with national trends, maternal mortality had remained comparatively high. Campbell and her colleagues regarded Huddersfield’s maternal and infant welfare arrangements as inadequate and voluntary antenatal notification with home visiting, in particular, as unsystematic and inefficient. Domiciliary visits tended to be perfunctory, seldom allowed for ‘anything approaching a complete physical examination’, and were not always welcome. The present system, Campbell wrote in her 1932 report, ‘may be excellent on paper but is very incomplete in practice’. The Ministry recommended the local authority establish a ‘properly equipped’ clinic for antenatal supervision. Officials found a recalcitrant council convinced that existing methods were satisfactory and unwilling to commit resources to building new facilities.^114^

During a national enquiry into maternal mortality in 1935, Ministry officials reasserted their concerns about antenatal care in Huddersfield. The proportion of notifications had leapt from 45 per cent to around 80 per cent in the five years since Gibson’s appointment. But the scheme remained, in the Ministry’s view, ineffective at reaching expectant mothers sufficiently early in pregnancy to permit adequate antenatal supervision. The MOH remained, like his predecessor, firmly committed to notification with visiting. Gibson robustly defended the Huddersfield system in correspondence with the Ministry, in annual reports, and in public lectures.^115^ He maintained that clinics would prove unpopular, and that the present scheme guaranteed expectant mothers received attention from qualified medical women. Notification of pregnancy, Gibson claimed, had been comparatively successful at reaching expectant mothers (the national average attendance at antenatal clinics was an estimated 42 per cent), while visiting was more cost effective than investing in bricks and mortar. Finally, he insisted that maternal deaths tended to occur among unnotified mothers who received no antenatal care at all.^116^ Ministry officials were assuaged and, though still unconvinced by notification, wished to avoid undermining the position of a reforming MOH who was demonstrably improving municipal services.^117^ More cordial relations with the HVNA, which took on all domiciliary midwifery work in Huddersfield after the 1936 Midwives Act, consolidated the trend. By the end of the Second World War, Gibson could boast that around 95 per cent of pregnancies in the borough were notified annually.^118^

Promoted by two successive MOHs over 35 years, Huddersfield’s formal notification of pregnancy scheme was exceptional. Only one other local authority, the metropolitan borough of Holborn, developed anything comparable in scope.^119^ But while the Huddersfield approach was recognised as unorthodox, municipal authorities were by the 1920s experimenting with a range of measures to extend the reach of antenatal services, many of which differed from notification in only subtle ways.

Some councils offered inducements directly to pregnant women to use antenatal clinics. Five London boroughs adopted ‘virtual notification of pregnancy’ in the interwar years by inviting expectant mothers to apply for clothing, free meals or food coupons.^120^ Other authorities concentrated on improving cooperation with midwives. Such strategies included inviting midwives to undertake antenatal care in municipal clinics under supervision, providing medical supplies and record forms, and offering compensation for either lost clientele or work undertaken on the authority’s behalf.^121^ In Bedfordshire county council’s antenatal scheme, for instance, midwives were paid five shillings ‘for every case in which they undertake and discharge certain well-defined duties’.^122^ Rather than institute such direct forms of surveillance as notification, the more orthodox approach was to promote awareness and acceptance of antenatal services gradually, through education and familiarity with local municipal staff. The importance of the health visitor in building ‘close intimacy’ with individual families was particularly emphasised, a strategy that MOHs could refer to as a ‘modified form of notification of pregnancy’.^123^

For all the suspicion of notification, as much communication between local officials, health and welfare workers and the voluntary sector took place on an ad hoc, ‘need-to-know’ basis as through formal record-keeping. This was perhaps especially true of antenatal care offered to lone mothers hostelised in ‘rescue homes’, and pregnant women suspected of having, or found to have, a venereal disease. Securing treatment for expectant mothers was a priority for the emerging interwar VD clinic network, yet was complicated by local officials’ concern to shield married women from stigma. Reaching these mothers relied on tacit knowledge, word-of-mouth referrals and personal relationships between institution staff.^124^

In Huddersfield, for example, there was ‘practically no [formal] cooperation between the maternity and infant welfare and VD departments, because officials worried this would discourage public acceptance of municipal antenatal services and wanted to protect married women from the imputations that might have accompanied attendance at the VD clinic. However, the matron of the local rescue home made it a ‘routine procedure to send every new admission to the [VD] clinic for examination’. The clinic also received for treatment ‘a certain number of expectant mothers’ from ‘outside practitioners’ (private GPs and midwives), and even some cases ‘unofficially’ from maternal and infant welfare staff.^125^ Institutional links between antenatal departments at maternity hospitals, VD clinics and the rescue sector blurred the lines between the surveillance of pregnancy and the control of venereal diseases within the maternity and infant welfare network.^126^

The steady, if protracted, expansion of antenatal care during the 1920s and 1930s, then, proceeded through a range of strategies, both formal and informal, for ‘linking up’ expectant mothers—married and unmarried—with medical supervision. These combined and evolved in different ways in different settings, and only local studies can capture this variety and complexity. Huddersfield’s notification of pregnancy scheme was unique. But since there was often slippage between ‘notification’ and its alternatives, and such measures were rarely publicised or rapidly abandoned, it is impossible to say definitively how many councils experimented with the practice. The progress or inhibition of such schemes hinged on the ambition and standing of the MOH, the internal dynamics of councils, and communication between service providers. In Huddersfield, as elsewhere, relations between health departments and midwives were paramount.

## Conclusion

Although experimentation with notification of pregnancy continued informally through the 1920s and 1930s, few schemes survived beyond the First World War. According to one MOH, the practice ‘was tried … and failed; its repetition cannot be recommended’; for another, it was ‘practically a dead letter’. One London-based health official ruefully noted in 1917 that the measure was ‘too much in advance of public ideas’.^127^ Opponents attributed such failures to the objections of mothers themselves. But though midwives claimed to have attended meetings of ‘working mothers’ protesting against the notification of pregnancy, and such sentiments fit with broader patterns of resistance to the efforts of local authorities to intervene in the lives of the poor, direct evidence of popular attitudes is rare.^128^ Debates over the issue reveal more about perceptions of working-class sensibilities, and the ways in which these were mobilised in professional disputes, than those attitudes per se.

Resistance to notification reflected many agendas, including medical practitioners’ traditional antipathy to state interference in the private relations between doctor and patient. That the most robust opposition came from midwives is a reminder of the agency of this occupational group in wider deliberations over public health reform. Ultimately, the controversy stemmed from divergent interpretations of the powers of health authorities, and the position of the expectant mother, within a modernising local welfare system. Visions of administrative control advocated by proponents of notification clashed with conceptions of female citizenship and midwives’ professional authority. ‘It is necessary to occasionally remind the authorities’, grumbled a ‘certified midwife’ in the feminist periodical *The Common Cause*, that the pregnant woman is neither ‘drain nor dairy, and could not be inspected as such’; rather, she is a ‘normal human being, with opinions, tastes, fancies, and rights of her own.’^129^

The controversy helps, above all, to illuminate midwives’ attitudes to antenatal care as they negotiated the opportunities and challenges of professionalisation. Recent historical writing on midwifery has rightly questioned the assumption that such antagonisms as those documented here were the inevitable consequence of either a historic struggle over the spheres of male and female practitioners or state regulation after 1902. Processes of conflict and compromise, as Mooney and Crook have both recently underlined, nevertheless decisively shaped the various elements and activities comprising the ‘administrative machinery’ of an evolving public health bureaucracy.^130^ Officials emphasised that antenatal care not only depended upon, but was also defined by relationships with local midwives, who mediated between municipal services and the poorer mothers they desired to reach. Closer engagement with local health departments across the interwar period, and consolidated by the 1936 Midwives Act, formalised midwives’ responsibility for antenatal supervision. But, even then, both the Ministry of Health and the rules of the CMB spelled out that there was no obligation to notify pregnancy; the midwife’s ‘professional relations with her patients should remain undisturbed’.^131^

Like so many other state-funded health services well into the interwar period, antenatal provision continued to depend on local interpretation and policy making. The impetus for notification of pregnancy came from MOHs, keen to assert their authority within an increasingly complex health and welfare apparatus. Proponents complained that earlier infant welfare legislation had been a ‘golden opportunity’ missed and the absence of a national system for notifying pregnancy remained a ‘grievous anomaly’.^132^ As birthrate decline, criminal abortion, maternal mortality and the effectiveness of antenatal care re-emerged as concerns on the eve of the Second World War, health officials again and again raised the prospect of making pregnancy formally notifiable within the context of socialised medicine.^133^ For all the acknowledged practical and ethical problems, members of the public health profession were still lobbying for notification as a solution to these challenges in the 1950s.^134^

By this time, however, the extension of municipal midwifery provision and the concentration of maternity care in hospitals, accelerated by the arrival of the National Health Service, had reshaped the occupational landscape and created new conditions for the surveillance of pregnancy. The controversy over notification of pregnancy had centred on the invasion of family privacy by local government officials, at a time when the powers and responsibilities of the MOH were at their zenith. But under the NHS, GPs’ surgeries and especially hospitals displaced the municipal clinic as the main venues of statutory antenatal care, part of a broader deterioration of the influence and reach of local health departments. The Huddersfield notification scheme was eventually discontinued in 1951, by which time 88 per cent of deliveries in the borough took place in hospital.^135^ ‘Nearly all’ mothers in Britain by now received some form of antenatal supervision, the result of the transition to free, hospital-oriented health care, educational campaigns, and wartime and post-war maternity entitlements.^136^ Incremental reforms, rather than the administrative practice of notification, ultimately transformed women’s expectations about when, where and from whom to seek medical attention during pregnancy.

## Footnotes

1 C. Templeman, ‘The Control of the Ante-natal Factors in Infant Mortality’, in *Report of the Proceedings of the National Conference on the Prevention of Destitution* (London: P. S. King, 1911), 75.

2 On the innovations from the early twentieth century, see, inter alia: A. Davin, ‘Imperialism and the Cult of Motherhood’, *History Workshop Journal*, 1978, 5, 9–65; J. Lewis, *The Politics of Motherhood. Child and Maternal Welfare in England, 1900–1939 (*London: Croom Helm*,* 1980). The most comprehensive overview of concerns about citizenship and national vitality is I. Zweiniger-Bargielowska, *Managing the Body: Beauty, Health, and Fitness in Britain 1880–1939* (Oxford: Oxford University Press, 2010).

3
*Forty-fourth Annual Report of the Local Government Board (LGB), 1914–15* (Cd. 8085), 64–5.

4 J.W. Ballantyne, ‘Mother Welfare in Pregnancy and Infant Health’, *Edinburgh Medical Journal*, 1917, 18 [new series], 348–61, 354.

5 On wartime conditions and infant welfare: D. Dwork, *War is Good for Babies and Other Young Children: A History of the Infant and Child Welfare Movement in England 1898–1918* (London: Tavistock, 1987).

6 G. Mooney, ‘Public Health Versus Private Practice: The Contested Development of Compulsory Infectious Disease Notification in Late-nineteenth-century Britain’, *Bulletin of the History of Medicine*, 1999, 73, 238–67.

7 Dwork, *War is Good*, 139–41.

8 J. Welshman, *Municipal Medicine: Public Health in Twentieth-Century Britain* (Oxford: Peter Lang, 2000); S. Szreter, *Health and Wealth: Studies in History and Policy* (Rochester: University of Rochester Press, 2005), 281–341.

9 For municipal medicine and the ‘local welfare state’: A. Levene, M. Powell, J. Stewart and B. Taylor, *Cradle to Grave: Municipal Medicine in Inter-war England and Wales* (Oxford: Peter Lang AG, 2011), 20–8.

10 J. Lewis, ‘The Working-Class Wife and Mother and State Intervention, 1870–1918, in J. Lewis, ed., *Women’s Experience of Home and Family, 1850–1940* (Oxford: Blackwell, 1986).

11 Exemplary local studies of maternal and infant welfare include L. Marks, *Metropolitan Maternity: Maternal and Infant Welfare Services in Early Twentieth-Century London* (Amsterdam: Rodopi, 1996).

12 A. Oakley, *The Captured Womb: A History of the Medical Care of Pregnant Women* (Oxford: Blackwell, 1984). For an overview of critiques, see J. Greenlees and L. Bryder, eds, *Western Maternity and Medicine, 1880–1990* (London: Pickering and Chatto, 2013), introduction.

13 For a review of this literature, see P. Dale and K. Fisher, ‘Implementing the 1902 Midwives Act: Assessing Problems, Developing Services and Creating a New Role for a Variety of Female Practitioners’, *Women’s History Review*, 2009, 18, 427–52.

14 Mooney, ‘Public Health and Private Practice’; D. Porter and R. Porter, ‘The Enforcement of Health: The British Debate,’ in E. Fee and D. Fox, eds, *Aids: The Burdens of History* (Berkeley: University of California Press, 1988), 97–120; S. Lemar; ‘“The Liberty to Spread Disaster”: Campaigning for Compulsion in the Control of Venereal Diseases in Edinburgh in the 1920s’, *Social* *History* *of* *Medicine*, 2006, 19, 73–86; A. Ferguson, *Should a Doctor Tell? The Evolution of Medical Confidentiality in Britain* (Farnham: Ashgate, 2013); A.-H. Maehle, *Contesting Medical Confidentiality, Origins of the Debate in the United States, Britain and Germany* (Chicago: University of Chicago Press, 2016).

15 G. Mooney, *Intrusive Interventions: Public Health, Domestic Space, and Infectious Disease Surveillance in England, 1850–1914* (Rochester: University of Rochester Press, 2015); T. Crook, *Governing Systems: Modernity and the Making of Public Health in England, 1830–1910* (Berkeley: University of California Press, 2016).

16 A. Borsay and B. Hunter, eds, *Nursing and Midwifery in Britain Since 1700* (Basingstoke: Palgrave, 2012), chs 6 and 7.

17 A. Hanley, ‘Scientific Truth into Homely Language’: The Training and Practice of Midwives in Ophthalmia Neonatorum, 1895–1914’, *Social* *History* *of* *Medicine*, 2014, 27, 199–220.

18 For comparative overviews, see G. Bock and P. Thane, eds, *Maternity and Gender Policies: Women and the Rise of the European Welfare States, 1880–1950s* (London: Routledge, 1991); S. Koven and S. Michel, *Mothers of a New World: Maternalist Politics and the Origins of Welfare States* (New York: Routledge, 1993).

19 J.W. Ballantyne, ‘Antenatal Therapeutics’, *British Medical Journal*, 1899, i, 889–93; S. Al-Gailani, ‘Pregnancy, Pathology and Public Morals: Making Antenatal Care in Early Twentieth-Century Edinburgh’, Greenlees and Bryder, eds, *Western Maternity*, 31–46.

20 M. Bondfield, *The National Care of Maternity: A Scheme Put Forward as a Basis for Discussion* (London: Women’s Co-operative Guild, 1914).

21 Al-Gailani, ‘Pregnancy, Pathology and Public Morals’. On voluntary provision for expectant mothers, see P. Thane, ‘Visions of Gender in the Making of the British Welfare State: The Case of Women in the British Labour Party and Social Policy 1906–45’, in Bock and Thane, *Maternity and Gender Policies*, 93–118; and E. Ross, *Love and Toil: Motherhood in Outcast London, 1870–1918* (Oxford: Oxford University Press, 1993), 215–19.

22 G. Newman, *Infant Mortality: A Social Problem* (London: Methuen, 1906), 61–89.

23
*Forty-second Annual Report of the LGB*, 1912–13 (Cd. 6909), 90; LGB memorandum on ‘Ante-natal hygiene and infant welfare work’, The National Archives (subsequently TNA) ED24/1377. On Newsholme and infant welfare, J. Eyler, *Sir Arthur Newsholme and State Medicine, 1885–1935* (Cambridge: Cambridge University Press, 1997), 295–338.

24
*Forty-third Annual Report of the LGB*, 1913–14 (Cd. 7612), 19.

25 ‘Maternity and Child Welfare’, *BMJ*, 1916, i, 241–2; M. Scharlieb, *The Welfare of the Expectant Mother* (London: Cassell, 1919).

26 Dwork, *War is Good*; Eyler, *Arthur Newsholme*, 295–338.

27
*The British Journal of Nursing*, 1916, 57, 103.

28 A. Donald, ‘The Care of the Pregnant Woman’, *BMJ*, 1916, ii, 33–5.

29
*Nursing Notes*, 1917, 30, 183.

30
*Medical Officer*, 1916, 16, 273; J. J. Buchan, ‘Ante-natal and Neo-natal Mortality’, *The British* *Journal* *of* *Nursing*, 1919, 63, 142.

31
*Forty-third Annual Report of the LGB*, 1913–14 (Cd. 7612), 31–3. On the hospital almoner: G. C. Gosling, *Payment and Philanthropy in British Healthcare, 1918–1948* (Manchester: Manchester University Press, 2017), 93–104.

32
*Report on an Enquiry into the Work of Existing Ante-natal Centres* (London: National League for Health, Maternity and Child Welfare, 1925), 12–13.

33 M. O. Haydon, ‘Antenatal Care by Midwives’, *Nursing Notes*, 1915, 28, 34–6; Haydon, ‘How to Make Antenatal Work Attractive to Mothers’, *Maternity and Child Welfare*, 1925, 9, 148–50. On the interplay between privacy, ignorance and respectability in working-class women’s attitudes to sex and the body, see Simon Szreter and Kate Fisher, *Sex Before the Sexual Revolution: Intimate Life in England, 1918–1963* (Cambridge: Cambridge University Press, 2010), esp. 268–316.

34 C. Dyhouse, ‘Working-Class Mothers and Infant Mortality in England, 1896–1914’, in C. Webster, ed., *Biology, Medicine and Society 1840–1940* (Cambridge: Cambridge University Press, 1981), 73–98; L. McCray Beier, *For Their Own Good: The Transformation of Working-Class Health Culture, 1880–1970* (Columbus: Ohio State University Press, 2008), esp. 264–309.

35 Mooney, ‘Public Health Versus Private Practice’; Mooney, *Intrusive Interventions*, 40–65; Crook, *Governing Systems*, 209–16.

36 J. Burns, ‘The Relationship Between Medicine and Public Health’, *BMJ*, 1913, ii, 362–9.

37 J. Lane-Claypon, *The Child Welfare Movement* (London: G. Bell, 1920), 15.

38 The classic study of the Acts, which enabled doctors to inspect women suspected of prostitution, is J. Walkowitz, *Prostitution and Victorian Society: Women, Class, and the State* (Cambridge: Cambridge University Press, 1980).

39 See, inter alia: R. Davidson, *Dangerous Liaisons: A Social History of Venereal Disease in Twentieth-Century Scotland* (Amsterdam: Rodopi, 2000); P. Cox, ‘Compulsion, Voluntarism, and Venereal Disease: Governing Sexual Health in England after the Contagious Diseases Acts’, *Journal of British Studies*, 2007, 46, 91–115; A. Hanley, *Medicine, Knowledge and Venereal Diseases in England, 1886–1916* (Basingstoke: Palgrave, 2017).

40
*RC on Venereal Diseases. Appendix to first report of the commissioners*, 1914 (Cd. 7475), 310; 314; 404.

41
*Lancet*, 1915, 185, 184; ‘Memorandum on the Notification of Pregnancy’, May 1919 (TNA MH 81/46).

42
*RC on Venereal Diseases*, 310; 314; 404; A. Routh, ‘Ante-natal Hygiene: Its Influence Upon Infantile Mortality,’ *BMJ*, 1914, ii, 355–63; J. W. Ballantyne, ‘The Notification of Pregnancy’, *BMJ*, 1916, i, 594–5. For wider medical interest in ‘antenatal wastage’, see G. Davis, ‘Stillbirth Registration and Perceptions of Infant Death, 1900–60: The Scottish Case in National Context’, *Economic History Review*, 2009, 62, 629–54; and R. Elliot, ‘Miscarriage, Abortion or Criminal Feticide: Understandings of Early Pregnancy Loss in Britain, 1900–1950’, *Studies in History and Philosophy of Science: Biological and Biomedical Sciences*, 2014, 47B, 248–56.

43 Cox, ‘Compulsion, Voluntarism and Venereal Disease’, 108; E. Thomson, ‘Between Separate Spheres: Medical Women, Moral Hygiene and the Edinburgh Hospital for Women and Children’, in S. Sturdy, ed., *Medicine and the Public Sphere: Britain 1550–2000* (London: Routledge, 2002), 107–22.

44 A. White, ‘Maternity and Child Welfare Work’, *Medical Officer*, 1915, 14, 189–92.

45 Quotations from notes on ‘Ante-natal hygiene and infant welfare work’, TNA ED24/1377, 5 and C. Berkeley, ‘The Importance of Getting Medical Practitioners and Midwives to Co-operate with the Local Health Authorities’, *Proceedings of the Royal Society of Medicine*, 1917, 10, 52–62. For laboratory-based pregnancy-testing: see J. Olszynko-Gryn, ‘The Demand for Pregnancy Testing: The Aschheim-Zondek Reaction, Diagnostic Versatility, and Laboratory Services in 1930s Britain’, *Studies in History and Philosophy of Science: Biological and Biomedical Sciences*, 2014, 47B, 233–47.

46 Ballantyne, ‘The Notification of Pregnancy’, 594.

47 J. M. Munro Kerr, ‘Toxemias of Pregnancy and Their Effect Upon Maternal and Infantile Mortality’, *Report of the Proceedings of the English-speaking Conference on Infant Mortality* (London: National Association for the Prevention of Infant Mortality and for the Welfare of Infancy, 1913), 423.

48 Cox, ‘Compulsion, Voluntarism, and Venereal Disease’.

49 Ballantyne, ‘The Notification of Pregnancy’, 595.

50 Routh, ‘Ante-natal Hygiene’, 357; J. W. Ballantyne, ‘The Nature of Pregnancy’, *BMJ*, 1914, i, 349–55. For the endowment of motherhood: S. Pedersen, *Family, Dependence and the Origins of the Welfare State: Britain and France 1914–1945* (Cambridge: Cambridge University Press, 1993), 138–77.

51
*Report of the Departmental Committee on Sickness Benefit Claims under the National Insurance Act; Evidence*, Vol. 1, (Cd. 7688), 395 and 435; and Vol. 3 (Cd. 7690), 217.

52 S. Pedersen, ‘Gender, Welfare, and Citizenship in Britain during the Great War’, *The American Historical Review*, 1990, 95, 983–1006; Levene, et. al., *Cradle to Grave*, ch. 4.

53 On the status of the MOH *c*.1914, see Szreter, *Health and Wealth*, 281–341; Welshman, *Municipal Medicine*, 31–5.

54 S. G. Moore, ‘Infantile Mortality and the Relative Practical Value of Measures Directed to its Prevention’, *Lancet*, 1916, 187, 944–8.

55 F. Honigsbaum, *The Struggle for the Ministry of Health, 1914–1919* (London: Bell, 1970), 36.

56
*Lancet*, 1911, 177, 1223; Dwork, *War is Good*, 139–41.

57 Special report of Council on Maternity and Child Welfare Schemes, 1915; and letter from J. S. Forrest, 5 July 1918, *Minutes of the BMA Maternity and Child Welfare Sub-Committee*, vol. I, Wellcome Library (WL) SA/BMA/C.80; *Public Health*, 1917, 31, 144.

58
*BMJ*, 1916, ii, 280–1.

59
*BMJ* supplement, 1916, 6 May, 97.

60
*BMJ*, 1916, ii, 280–1.

61 White, ‘Maternity and Child Welfare Work’.

62
*BMJ* supplement, 1916, 6 May, 97.

63
*BMJ*, 1907, ii, 696.

64 Minutes of BMA Annual Representative Meeting, 28 July 1916, WL SA/BMA/J.128), 16.

65
*Supplement to the Forty-fourth Annual Report of the LGB, 1914–15* (Cd. 8085), 65–6.

66
*Forty-fifth Annual Report of the LGB, 1915–16* (Cd. 8423), 39.

67
*Supplement to the Forty-fourth Annual Report of the LGB,* 65–6; and *Forty-fifth Annual Report of the LGB*, 39.

68
*Annual Report of the Chief Medical Officer, 1920* (Cmd. 978), 111.

69 Dale and Fisher, ‘Implementing the 1902 Midwives Act’.

70
*Nursing Notes*, 1916, 29, 249–50.

71 B. V. Heagerty, ‘Willing Handmaidens of Science? The Struggle over the New Midwife in Early Twentieth-Century England’, in M. Kirkham and E. Perkins, eds, *Reflections on Midwifery* (London: Baillière Tindal, 1997), 70–95; J. Hannam, ‘Rosalind Paget: The Midwife, The Women’s Movement and Reform Before 1914’, in H. Marland and A. M. Rafferty, eds, *Midwives, Society and Childbirth: Debates and Controversies in the Modern Period* (London: Routledge, 1997), 81–101.

72 See Midwives Institute Executive Council and Advisory Committee Minute Books 1915–1921, Library of the Royal College of Obstetricians and Gynaecologists [RCOG] A1/19–21 and Representative Committee Minute Book, RCOG C2/2; Women Sanitary Inspectors Association (WSIA) Annual Report (1915–1916), WL SA/HVA/A.1/9, 17.

73 Minutes of the Midwives’ Institute Executive Council and Advisory Committee, 27 January 1916, RCOG A1/19, 36; and letter from Institute Secretary Paulina Ffynes Clinton, 1 Feb 1916 in RCOG A1/20; *Nursing Mirror*, 1916, 22, 443.

74 J. E. St. Clair, ‘Midwives’ Grievances’, *Lancet*, 1916, 187, 267.

75
*Nursing Notes*, 1918, 31, 99.

76
*Nursing Mirror*, 1917, 24, 324; *Common Cause*, 1917, 29 June, 154; *Nursing Notes*, 1918, 31, 64.

77
*The British* *Journal* *of* *Nursing*, 1917, 58, 17.

78 M. J. D. Roberts, ‘Feminism and the State in Later Victorian Britain’, *Historical Journal*, 1995, 38, 85–110.

79 E. Pethick Lawrence, ‘A New Menace to Women’, *Votes for Women*, 1913, 7 November, 81; C. Nina Boyle, ‘Treatment of Venereal Disease’, *The Vote*, 1916, 14, 1115. For early twentieth-century feminist views on VD policy: L. Bland, *Banishing the Beast: English Feminism and Sexual Morality, 1885–1914* (London: I.B. Tauris, 1995).

80
*Nursing Mirror*, 1918, 26, 354. For similar criticisms: A. E. R. ‘Health and the State’; *Nursing Notes*, 1917, 29, 236; A. Martin, ‘The Irresponsibility of the Father’, *The Nineteenth Century and After*, 1918, 84, 1091–6.

81 Midwives’ Institute Council Minute Book, 4 July 1919, RCOG A1/21, 11; *Nursing Mirror*, 1916, 23, 230; *Common Cause*, 1917, 29 June, 154.

82 Moore, ‘The Notification of Pregnancy’, 168.

83
*Nursing Notes* 1918, 31, 67; *Medical Officer*, 1916, 16, 273; 282.

84
*Common Cause*, 1917, 29 June, 154; A. E. R., ‘Health and the State,’ *New Age*, 1917, 21, 332–3.

85 D. Vincent, *The Culture of Secrecy: Britain, 1832–1998* (Oxford: Oxford University Press, 1998), ch. 4. See also Vincent, *Poor Citizens: The State and the Poor in Twentieth-Century Britain* (London: Longman, 1991), 75–9 on privacy as a resource for the poor; and Ross, *Love and Toil*, 206–9 on resistance to the ‘invasion’ of the working-class home.

86
*Common Cause*, 1917, 29 June, 154.

87
*Nursing Notes*, 1918, 31, 99.

88 Z. Scruby, ‘Ante-natal Care’, *Nursing Notes*, 1917, 30, 203.

89 R. Paget, ‘Supervision of Midwives’, *The Times*, 1916, 2 November, 9.

90
*Nursing Notes*, 1917, 30, 111–14.

91 A. E. R. ‘Health and the State’; *The Midwife*, 1917, 58, 17.

92 Midwives’ Institute Council Minute Book, 4 July 1919, RCOG A1/21, 11

93
*Nursing Notes*, 1918, 31, 36.

94 F. Champneys, ‘Health Visitors and Birth Inquiry Cards’, *BMJ*, 1916, ii, 438; CMB Board Minutes TNA DV1/8, pp. 190, 243, and 266.

95 Hannam, ‘Rosalind Paget’; T. McIntosh, *A Social History of Maternity and Childbirth: Key Themes in Maternity Care* (London: Routledge, 2012), ch. 3.

96 See correspondence between Fletcher and Robert Morant, the first Permanent Secretary to the new Ministry of Health, relating to the ‘Memorandum on the Notification of Pregnancy’, May 1919, TNA MH 81/46.

97
*Fifteenth Annual Report of the Ministry of Health 1933–1934* (Cmd. 4664), 82. On mothercraft and the role of women’s organizations: see C. Beaumont, *Housewives and Citizens: Domesticity and the Women’s Movement in England, 1928–64* (Manchester: Manchester University Press, 2013), 101–34.

98 S. M. Marsters, ‘Scheme for the Working of Maternity and Child Welfare Services’, *The Midwife*, 1917, 59, 63–4. See C. Davies, ‘The Health Visitor as Mother’s Friend: A Woman’s Place in Public Health, 1900–14’, *Social* *History* *of* *Medicine*, 1988, 1, 39–59 for the interplay between class and the vocabulary of ‘friendship’ in health visiting around 1900.

99
*Maternal Mortality in Childbirth: Ante-natal Clinics: Their Conduct and Scope.* Ministry of Health Memorandum 145/MCW (London: HMSO, 1929); *Ministry of Health Report on an Investigation into Maternal Mortality* (Cmd. 5422), 248–9.

100 A. F. G. Spinks, ‘Maternity and Child Welfare Practice: A Criticism’, *Journal* *of the* *Royal* *Sanitary* *Institute*, 1925, 45, 409–11.

101 H. Harvey Evers, ‘Ante-natal Work’, *Public Health*, 1927, 40, 51–5.

102 H. Marland, ‘A Pioneer in Infant Welfare: The Huddersfield Scheme 1903–1920’, *Social* *History* *of* *Medicine*, 1993, 6, 25–50.

103 Huddersfield Borough Health Committee Minutes, 1916–18, West Yorkshire Archive Service, Kirklees [WYAS], KMT18/12/2/38/25, 15.

104 S. G. Moore, ‘The Notification of Pregnancy’, *Journal of the Royal Sanitary Institute*, 1920, 40, 165–8; Moore, ‘Five Years’ Experience of the Notification of Pregnancy’, *Public Health*, 1921, 35, 183–5.

105 S. G. Moore, ‘Ante-natal Hygiene and its Bearing on Neo-natal Mortality’, *Journal of the Royal Sanitary Institute*, 1923, 44, 373.

106 Moore, ‘The Notification of Pregnancy’.

107 Huddersfield Borough Health Committee Minutes, 1916–18, WYAS KMT18/12/2/38/25, 215; ‘Special Report on Maternity and Child Welfare Work’, appendix to *Huddersfield MOH, Annual Report for 1932*, 113.

108 Public Health Survey: Huddersfield; Survey Report, NA MH/66/680.

109 By the early 1930s, the HVNA was responsible for around two-thirds of midwifery cases in the borough: *Huddersfield MOH, Annual Report for 1937*, 53.

110 Huddersfield Victorian Nurses Association Minute Book, vol. 1, West Yorkshire Archive Service, Wakefield C347/1/2, reports on meetings on 18 January 1916, 15 February 1916 and 29 April 1920.

111
*Huddersfield MOH, Annual Report for 1929*, 28.

112
*Huddersfield MOH, Annual Report for 1933*, 41.

113 On the public health survey, see Levene et al., *Cradle to Grave*, 34.

114 Public Health Survey: Huddersfield; Survey Report and Appendices, NA MH/66/680 and NA MH/66/681.

115 Public Health Survey: Huddersfield; Correspondence, NA MH/66/682.

116 J. M. Gibson, ‘The Huddersfield Scheme for Maternity and Child Welfare Work’, *Journal of the Royal Sanitary Institute*, 1934, 55, 491–506.

117 Public Health Survey: Huddersfield; Correspondence, NA MH/66/682. For the Ministry’s ‘hands-off advisory relationship’ with local authorities, see B. Taylor, J. Stewart and M. Powell, ‘Central and Local Government and the Provision of Municipal Medicine, 1919–39’, *English Historical Review*, 2007, 122, 397–426.

118
*Public Health*, 1936, 49, 367; *Huddersfield MOH, Annual Report for 1951*, 5.

119 Holborn’s notification of pregnancy scheme was launched in 1927 and survived until 1948: *Report of the MOH for the London Borough of Holborn, 1927* (London: Diprose, Bateman and Co., 1928), 99.

120 ‘Ante-natal Work at Centres for Expectant Mothers’, *Public Health*, 1925, 39, 29–30.

121 J. Robertson, ‘The Welfare of the Expectant Mother’, *Medical Officer*, 1923, 30, 209.

122
*Report of the MOH for Bedfordshire County Council, 1925* (Bedford: C.F. Timaeus, 1926), 49.

123
*Report of the MOH for the City and Royal Burgh of Edinburgh, 1928* (Leith: Mackenzie and Storrie, 1929), 55.

124 Cox, ‘Compulsion, Voluntarism and Venereal Disease’, 108.

125 Public Health Survey: Huddersfield; Survey Report, NA MH/66/680.

126 Davidson, *Dangerous Liaisons*, 109.

127 Sidney Davies, MOH of Woolwich in *Nursing Notes*, 1917, 30, 148; *Report of the MOH for the County Borough of Dewsbury, 1918* (Dewsbury: S. Dawson, 1919), 38; *Report of the MOH for the Urban District of Barking Town, 1919* (Ilford: Recorders Ltd, 1920), 31.

128
*Nursing Notes*, 1918, 31, 99.

129
*Common Cause*, 1917, 29 June, 154.

130 Mooney, *Intrusive Interventions*, 40–68; Crook, *Governing Systems*.

131
*Annual Report of the Chief Medical Officer, 1920* (Cmd. 978), 111; Appended Leaflet on Antenatal Care, *Rules Framed by the Central Midwives Board under the Midwives Acts 1902 to 1936*, 14th edition (London: Central Midwives Board, 1939), 110–15.

132 Spinks, ‘Maternity and Child Welfare Practice’, 409–10.

133
*Lancet*, 1935, 226, 159–60; *Lancet*, 1937, 230, 1340; W. G. Booth, ‘Social Medicine’, *Public Health*, 1943, 56, 80–2.

134 Scottish Health Services Council, *Maternity Services in Scotland* (Edinburgh: Department of Health for Scotland, 1959), 17.

135
*Huddersfield MOH, Annual Report for 1952*, 15.

136 F. J. Browne and J. C. McClure Browne, *Antenatal and Postnatal Care* (London: Churchill, 1955), 12; Oakley, *Captured Womb*, 135–7; Beaumont, *Housewives and Citizens*, 106–21.

## Notes

The author is a teaching associate at the Department of History and Philosophy of Science, University of Cambridge. He is a contributor to the Wellcome Trust-funded strategic award project on the theme ‘Generation to Reproduction’, which supported the work for this article. His research focuses on the history of pregnancy and childbirth from the late nineteenth century to the present day.

